# Multi-Scale Investigation of Fly Ash Aggregates (FAAs) in Concrete: From Macroscopic Physical–Mechanical Properties to Microscopic Structure of Hydration Products

**DOI:** 10.3390/ma18112651

**Published:** 2025-06-05

**Authors:** Xue-Fei Chen, Xiu-Cheng Zhang, Ying Peng

**Affiliations:** 1 School of Civil Engineering, Putian University, Putian 351100, China; 2 Engineering Research Center of Disaster Prevention and Mitigation of Southeast Coastal Engineering Structures (JDGC03), Fujian Province University, Putian 351100, China

**Keywords:** fly ash, solid waste, aggregate, concrete

## Abstract

Fly ash aggregates (FAAs) were synthesized via a hydrothermal process, involving the reaction of fly ash and cement at 180 °C under saturated steam conditions. A thorough examination was carried out to evaluate the impact of cement content on the physico-mechanical properties of the resulting FAAs. A comprehensive exploration was undertaken to decipher the mechanisms by which cement modulates the cylinder compressive strength of FAAs, encompassing mineralogical composition, microstructure, insoluble residue content, and loss on ignition. As the cement proportion increased, a concomitant rise in the amount of hydration products was observed, leading to an enhanced filling effect. This, subsequently, resulted in reduced water absorption and increased apparent density of the FAAs. The augmented filling effect of hydration products contributed to a gradual elevation in the cylinder compressive strength of FAAs as cement content escalated from 5 to 35 wt%. However, a significant transition occurred when cement content surpassed 35%, reaching 35–45 wt%. Within this range, the micro-aggregate effect was diminished, causing a decrease in cylinder compressive strength. The optimal equilibrium between the filling effect and micro-aggregate effect was attained at 35 wt% cement content, where the cylinder compressive strength of FAAs reached its peak value of 18.5 MPa. This research is expected to provide a feasible approach for solid waste reduction, with a particular emphasis on the utilization of fly ash.

## 1. Introduction

Fly ash (FA), an industrial byproduct of coal combustion in power plants, poses significant environmental challenges. The burning of one ton of coal generates approximately 250 to 300 kg of FA, amounting to an annual production of up to 160 million tons. As of 2020, China’s total FA stockpile has escalated to 3 billion tons, occupying vast land resources and causing direct pollution of groundwater, air, and soil [1,2,3]. This accumulation represents a significant source of solid waste pollution in our country, impeding resource utilization efficiency and hindering the necessary green transformation for socio-economic development. Aligned with the national “dual carbon” strategy, promoting the utilization of FA is paramount for facilitating a green societal transition.

FA finds primary application in concrete as a typical supplementary cementitious material [4]. However, the technological advancement in its comprehensive utilization remains rudimentary, limiting the value-added potential of these products. Research efforts have focused on exploring high-value applications of FA, such as wastewater treatment, flue gas purification, extraction of alumina, silica, and rare metals, as well as the synthesis of ceramic and zeolite molecular sieves. The production of lightweight aggregates (LWAs) from FA emerges as a pivotal approach in promoting circular economy principles and expanding its diversified utilization channels. LWAs, characterized by their low density, high strength, and porosity, offer distinct advantages. Their use as a partial or complete replacement for traditional river sand and crushed stone in the preparation of lightweight aggregate concrete enhances durability and seismic resistance while reducing the product’s self-weight [5,6,7]. This, in turn, diminishes carbon emissions during both the construction and operational phases of buildings. Consequently, LWAs exhibit substantial market competitiveness in applications such as prefabricated structures, long-span bridges, and high-rise buildings.

Due to the scarcity of natural LWAs resources, artificial LWAs have gained prominence. These high-quality materials can be tailored to meet specific performance requirements, with adjustable formulations and production processes. Artificial LWAs can be manufactured through sintering or non-sintering methods [8,9,10]. Although sintered LWAs currently dominate the market, they are associated with high energy consumption and relatively low yields, stemming from inadequate initial research investments. Additionally, these products often exhibit suboptimal performance and rely heavily on limited resources like clay. With advancements in prefabricated construction technologies and increasing demand for LWAs, innovative products with superior performance have recently emerged. These include the substitution of shale and silt for clay or the utilization of solid waste through compositional design to produce high-performance sintered LWAs [11,12]. However, traditional sintering processes, requiring temperatures exceeding 1000 °C, result in significant energy consumption, posing challenges to environmental and energy conservation efforts. It has become increasingly difficult to obtain approval for investments involving the use of clay or similar resources in sintering processes.

In contrast, non-sintered LWAs primarily utilize cement-based materials as binders and incorporate various industrial waste products as raw materials, significantly reducing material costs. These processes do not require high-temperature sintering, thereby substantially lowering energy consumption, aligning with sustainable development strategies. Furthermore, the formulation and production techniques of non-sintered LWAs are more adaptable to design modifications, facilitating compliance with specific performance criteria. Given their shared origin with concrete, non-sintered LWAs exhibit superior compatibility, yielding favorable economic, environmental, and social benefits. Autoclaved lightweight aggregate, produced using calcareous and siliceous raw materials through hydrothermal synthesis curing technology, represents a type of non-sintered aggregate [9,13,14]. Fly ash serves as a commonly utilized siliceous raw material in the production of autoclaved products. The primary chemical components within FA, namely SiO_2_ and Al_2_O_3_, react under high temperature and pressure with Ca^2+^ in solution to form hydrated calcium silicate or hydrated calcium aluminate, imparting excellent mechanical properties to autoclaved products. However, the current landscape is predominantly focused on autoclaved bricks and autoclaved aerated concrete, with limited research on autoclaved lightweight aggregates. Despite notable progress by Ma et al. [15] in preparing autoclaved lightweight aggregates using propylene oxide slag and FA, the exploration of variation laws and influencing mechanisms on the physical and mechanical properties of autoclaved fly ash aggregates (FAAs) remains unfinished.

Herein, a comprehensive evaluation of various properties of the FAAs, including apparent density, cylinder compressive strength, water absorption, mineral composition, microstructure, and loss on ignition, is conducted. This assessment aims to elucidate the influence and mechanisms by which cement affects the physical and mechanical properties of autoclaved FAAs. Ultimately, this study strives to provide a theoretical foundation and technical support for the application of fly ash in autoclaved aggregate production, paving the way for more sustainable and environmentally friendly construction practices.

## 2. Experimental Details

### 2.1. Materials

Fly ash (FA) and ordinary Portland cement (OPC, grade 52.5) were employed in the manufacture of fly ash aggregates (FAAs). The FA was sourced from a power plant located in Hefei, China, while the OPC was procured from Jiangnan Onoda Cement Co., Ltd., Jiangnan, China. Table 1, obtained from the XRF (S8 Tiger, Bruker, Billerica, MA, USA), presents the primary chemical constituents of both the cement and FA. The average particle size (D50) of the FA was determined to be 6.98 mm via a particle size (Mastersizer 3000, Malvern Panalytical, Almelo, The Netherlands), indicating its fine nature, thus rendering it suitable for direct utilization in the production of FAAs.

### 2.2. Sample Preparation

**Figure 1:**
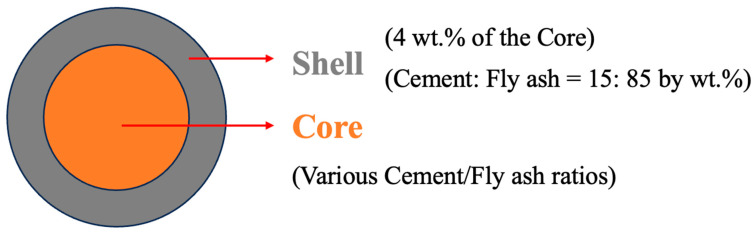
Schematic diagram of fly ash aggregates (FAAs).

Fly ash aggregates (FAAs) comprise two principal constituents: the shell and the core, as depicted in Figure 1. Prior research has demonstrated that the shell thickness accounts for 4% by weight of the core. The shell is composed of fly ash (FA) and cement in a mass ratio of 85:15, with all aggregates possessing an identical shell composition. The core consists of both cement and FA, with its total mass summing to 100%. To investigate the variations in the physical and mechanical properties of autoclaved FAAs, cement was systematically substituted for FA at regular intervals of 5% by mass. Among these formulations, the reference group was comprised 5% cement and 95% FA, designated as C5F95. A comprehensive mix proportion design for FAAs is presented in Table 2.

The core materials were mixed and subsequently transferred into a self-designed pelletization disc. Water, constituting 20–30 wt% of the total dry mass of the core materials, was sprayed to facilitate the formation of pellets. The pellets were allowed to grow to a size range of 5–16 mm. Thereafter, the shell powder mixture was uniformly applied onto the pellets to create green balls. These green balls underwent natural curing at ambient temperature for a minimum duration of 24 h, prior to being transferred to a fast-opening rotary stirring reactor for hydrothermal synthesis curing. The hydrothermal synthesis curing protocol employed in this experiment entailed the following steps. Initially, the temperature was increased from room temperature to a curing temperature of 187 °C over a period of 3 h. Subsequently, both the temperature and pressure were maintained at 187 °C and 1 MPa, respectively, for a duration of 10 h. Following this, a gradual cooling process was initiated at a rate of 2 °C per hour, until room temperature was attained within two hours post-curing. Upon completion of the curing process, the FAAs granules were extracted and placed in an oven set at 105 °C, where they were dried until a constant weight was achieved. Finally, the physical and mechanical properties of the FAAs were subjected to rigorous testing.

### 2.3. Testing

The physical attributes of fly ash aggregate (FAAs), encompassing apparent density, water absorption capacity, and cylinder compressive strength, were ascertained per the GB/T 17431.2 standard [16], utilizing calculations denoted by Equations (1)–(4). The weighed dry FAA specimens (m) underwent immersion in water for 1 h, intending to mitigate the impact of water absorption by the aggregates on the determination of apparent density. Subsequently, the specimens were processed to achieve a saturated surface-dry condition using damp towels. Thereafter, the aggregates were introduced into a graduated cylinder, followed by the addition of an additional 500 cm^3^ of water, and the final volume (V) of the graduated cylinder was recorded. The apparent density of the aggregates was subsequently derived based on the formulation outlined in Equation (2).

(1) $$\rho_{2}=\frac{10^{3}\times m}{V-500}$$

where $\rho_{2}$ is the apparent density (kg/m^3^); m is the dry mass of aggregates (g); V is volume of the final value of the graduated cylinder (cm^3^); 500 is the volume of additional water in the graduated cylinder (cm^3^).

Water absorption is the mass difference between the dry aggregates and water-saturated aggregates (immersed in water for 1 h and 24 h). The amounts after 1 h water absorption and 24 h water absorption were calculated as Equation (3).

(2) $$W_{i}=\frac{100\%\times \left(m_{i}-m\right)}{m}$$

where $W_{i}$ is the water absorption of aggregates (%), i = 1 is 1 h water absorption (%), i = 24 is the 24 h water absorption (%); m is the dry mass of aggregates (g); $m_{i}$ is the mass of aggregates after immersion in water (g),$\;m_{1}$ is the mass immersed in water for 1 h (g), $m_{24}$ was the mass of aggregates immersed in water for 24 h (g).

Aggregates possessing diameters ranged from 5 to 16 mm, with a size of 70 wt% in the 10–16 mm range. Subsequently, by DYE-2000, a load was applied uniformly upon the aggregates. The magnitude of the pressure was diligently recorded at the juncture when the depth of punch indentation reached 20 mm. This recorded value serves as an indicator of the aggregates’ strength, which is denominated as the cylinder compressive strength and is computed in accordance with Equation (4).

(3) $$f=\frac{P}{A}$$

where f is the cylinder compressive strength (MPa); P is the recorded pressure when the depth of punch indentation was 20 mm (N); A is the area of a cylinder with inner diameter of 56.9 mm and height of 120 mm (mm^2^).

The loss on ignition of FAAs is determined according to the standard JC/T 478.2 [17].

(4) $$\mathrm{LOI}=\frac{m_{2}{-m}_{0}}{m_{1}}\times 100$$

where LOI is the loss on ignition content of samples (%); $m_{0}$ is the mass of the crucible (g); $m_{1}$ is the mass of the sample before ignition (g); $m_{2}$ is the total mass of the sample and crucible after ignition (g).

The insoluble matter content of samples was tested according to GB/T 12960 [18] and it was calculated as Equation (6).

(5) $$R=\frac{100\%\times m_{2}}{m_{1}}$$

where R was the insoluble matter content of samples (%); $m_{1}$ was the initial mass of samples (g); $m_{2}$ was the mass of samples after acidification and calcination (g).

The crystalline phase composition of the samples was characterized through X-ray diffraction (XRD) analysis, employing a Model D8 advance instrument from Bruker, Germany, with CuKa radiation (wavelength, k = 1.542 Å) operated at 40 kV. The analysis was performed over a 2θ scanning range of 5° to 65°, with a step size of 0.02° and a time per step of 2 s. These parameters were selected to ensure adequate resolution for identifying crystalline phases while maintaining a reasonable analysis time. Main JCPDS/ICDD PDF cards used for indexing the peaks are PDF# 83-1520, PDF# 89-6451, PDF# 46-1045, PDF# 15-0776, PDF# 43-0118, and PDF# 33-0306. The microstructural features were observed using a scanning electron microscope (SEM), specifically the Model Quant 250FEG from FEI, Netherlands. To enhance the image quality, the surface of the samples was subjected to sputter coating with a gold layer.

## 3. Results and Discussion

### 3.1. Macro Physico-Mechanical Properties of Fly Ash Aggregates (FAAs)

#### 3.1.1. Water Absorption

**Figure 2:**
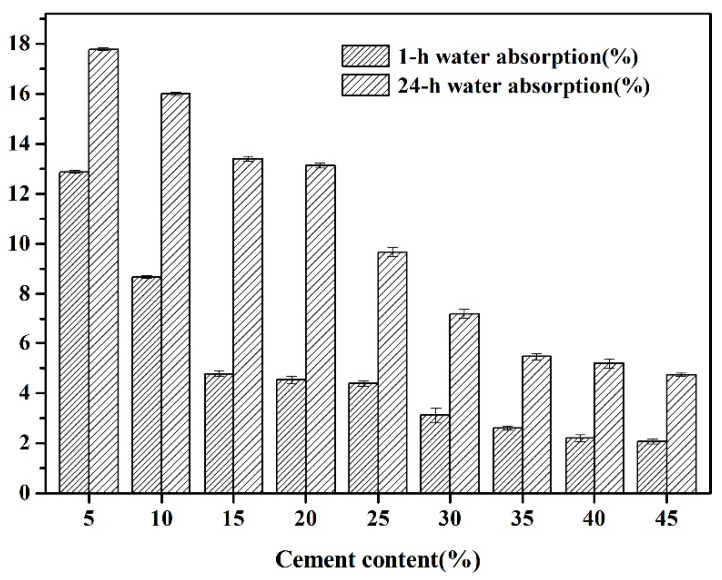
Amounts after 1 h and 24 h water absorption of fly ash aggregates (FAAs) of C5F95–C45F55.

As illustrated in Figure 2, the water absorption capacity of fly ash-based autoclaved aerated aggregates (FAAs) exhibits a decreasing trend at both 1 h and 24 h intervals with the augmentation of cement content. This phenomenon can be ascribed to the filling effect exerted by the hydration products generated during the autoclaved hydrothermal curing process. As the cement content escalates, the quantity of these hydration products also increases, thereby amplifying the filling effect and consequently enhancing the density of FAAs.

FA is characterized as a material with limited plasticity [19,20]. In instances where the proportion of FA is high and the cement content is low, the plasticity of the raw FAAs diminishes, leading to a compromised bonding capacity among the materials. This results in an elevated number of pores and voids within the raw FAAs, ultimately detracting from the density of the final product, FAAs. Consequently, the FAAs specimen designated as C5F95 (with a maximum fly ash content of 95 wt% and a cement content of 5 wt.%) demonstrates the highest water absorption rates: 12.88% at 1 h and 17.70% at 24 h. In comparison to C5F95, the FAAs specimens C10F90, C15F85, C20F80, C25F75, C30F70, C35F65, C40F60, and C45F55 exhibit reductions in water absorption at 1 h by approximately 32.7%, 62.8%, 64.8%, 65.8%, 75.7%, 79.8%, 82.9%, and 83.9%, respectively; the corresponding reductions at 24 h are approximately 10.1%, 24.7%, 26.2%, 45.6%, 59.5%, 69.3%, 70.8%, and 73.4%, respectively. These findings reveal that the incorporation of cement into FAAs effectively diminishes their water absorption capacity. Moreover, it is evident that as the cement content is incremented, this reduction in water absorption becomes increasingly significant. Zhu et al. [21] provide corroborative evidence, asserting that lightweight aggregates (LWAs) exhibiting high water absorption continue to absorb moisture even at relatively low saturation level. This persistent moisture absorption results in an escalation of both slump loss and time-dependent losses within LWAs concrete mixtures. Consequently, by augmenting the cement content to lower the water absorption of FAAs, it is possible to enhance the slump performance and simultaneously ameliorate the time-dependent losses in such concrete mixtures.

#### 3.1.2. Apparent Density

**Figure 3:**
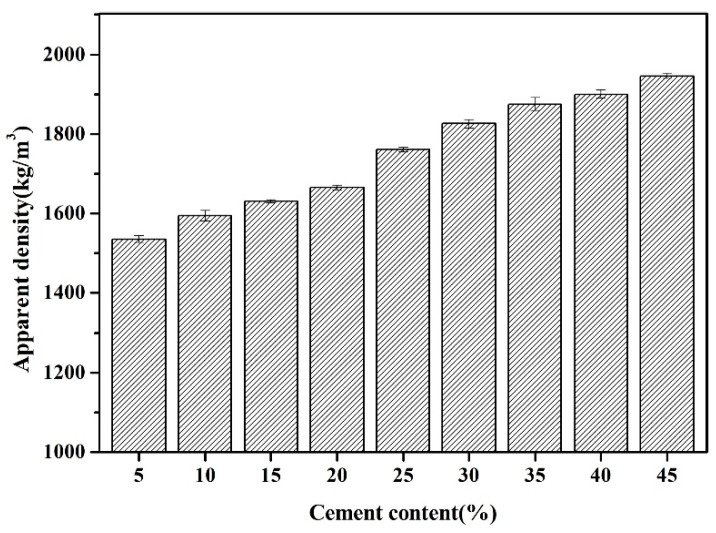
Apparent density of FAAs C5F95–C45F5.

The apparent density of FAAs, spanning from C5F95 to C45F55, is graphically represented in Figure 3. It is discernible that with the progressive increment of cement content from 5% to 10%, 15%, 20%, 25%, 30%, 35%, 40%, and ultimately 45%, the density of the FAAs escalates from 1536 kg/m^3^ to 1595 kg/m^3^, 1631 kg/m^3^, 1666 kg/m^3^, 1761 kg/m^3^, 1826 kg/m^3^, 1875 kg/m^3^, 1900 kg/m^3^, and culminates at 1946 kg/m^3^. This augmentation in apparent density can be attributed to the heightened content of hydration products and an augmented filling effect, which synergistically enhance the compactness and elevate the apparent density of the FAAs. As illustrated in Figure 3, when the cement content is 5 wt% and the fly ash content is 95 wt.%, the FAA C5F95 exhibits a minimum apparent density of 1536 kg/m^3^, which is slightly higher than some types of lightweight aggregates, such as expanded shale ranging 800–1200 kg/m^3^ [22] and pumice ranging 500–900 kg/m^3^ [23]. The advantages of utilizing low-density LWAs in concrete, such as diminishing the self-weight of concrete structures, reducing cross-sectional dimensions, conserving reinforcement materials, and decreasing costs related to foundation and substructure treatments, are corroborated by Tanyildizi et al. [24].

Moreover, it is noteworthy that the utilization rate of FA in C5F95 is as high as 95%, whereas that of cement is merely 5%. Cement production is a significant contributor to CO_2_ emissions, accounting for approximately 7% [25,26]. Consequently, the utilization of a minimal amount of cement is pivotal in mitigating CO_2_ emissions. Furthermore, given that FA constitutes a substantial volume of industrial solid waste, its recycling also plays a vital role in emission reduction and environmental preservation. Hence, the utilization of low cement content coupled with high fly ash content in FAAs holds immense importance. The apparent density of the FAAs was found to vary between 1536 and 1946 kg/m^3^, as depicted in Figure 3. Following the specifications delineated in GB/T 17431.1 [27], particles with an apparent density less than 2000 kg/m^3^ are categorized as LWAs. Notably, the apparent densities of all the examined FAAs fall below this 2000 kg/m^3^ threshold, thereby fulfilling the criteria for lightweight materials.

#### 3.1.3. Cylinder Compressive Strength

**Figure 4:**
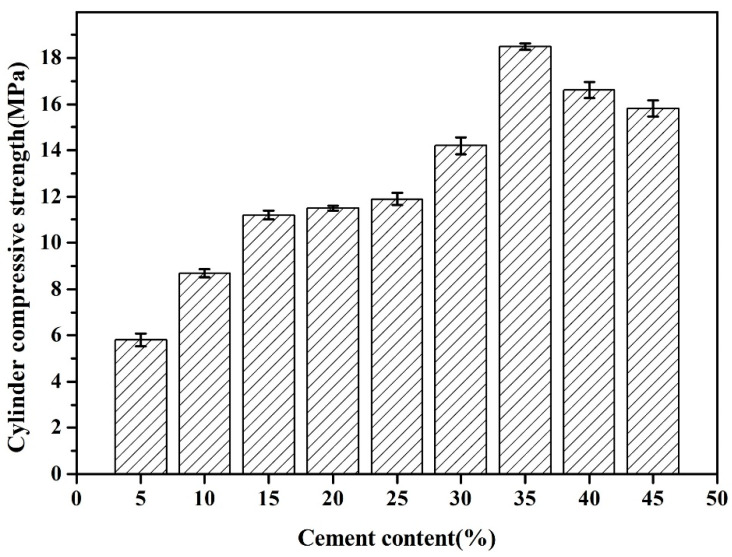
Cylinder compressive strength of FAAs.

Results depicted in Figure 4 elucidate the cylinder compressive strength of FAAs. It is manifest that the cylinder compressive strength exhibits an initial ascent followed by a descent with an escalation in cement content. As the cement content increases from 5% to 35% by weight, the axial compressive strength of the FAAs rises from 5.8 MPa to a maximum of 18.5 MPa at a cement content of 35%. Nonetheless, a further increase in cement content from 35% to 45% results in a decline in cylinder compressive strength to 15.8 MPa. Consequently, it can be inferred that the optimal cylinder compressive strength is attained at a composition designated as C35F65.

FAAs are synthesized within an autoclaved CaO-SiO_2_-Al_2_O_3_-H_2_O system under hydrothermal conditions, wherein Ca(OH)_2_, SiO_2_, and Al_2_O_3_ undergo reactions with H_2_O to form C-S-H and C-A-H. At lower cement incorporation levels, the generation of hydration products is inadequate, leading to feeble filling effects and consequently, loose connections between fly ash particles and localized structural porosity. Hence, it is imperative to employ an appropriate cement content to attain the optimal compressive strength values for FAAs. The formation of hydration products, as substantiated by XRD, SEM, and insoluble matter investigations, is presented in the following section.

In comparison to C5F95, the cylinder compressive strength of FAAs C10F90, C15F85, C20F80, C25F75, C30F70, C35F65, C40F60, and C45F55 exhibits increments of approximately 50%, 93%, 98%, 105%, 145%, 219%, 186%, and 172%, respectively. This underscores the significant enhancement in cylinder compressive strength of FAAs with increasing cement content. Notably, the enhancement in cylinder compressive strength reaches its zenith at an impressive rate of 219% when the cement content attains 35%. Research has corroborated that, with identical cement mortar, the mechanical properties of LWAs constitute a pivotal determinant of concrete strength. Thus, higher strength in LWAs correlates with enhanced overall strength of concrete. Consequently, augmenting cement content to elevate the strength of FAAs holds substantial importance for enhancing the strength of lightweight aggregate concrete.

The cement industry is characterized by substantial energy consumption and considerable pollution. The production of one ton of cement necessitates copious amounts of raw materials, including coal and limestone, resulting in the emission of approximately 0.8 tons of CO_2_ per ton of cement produced. Furthermore, this process emits various toxic gases (such as NOx and SOx), heavy metals, and PM2.5 particulates. As illustrated in Figure 4, FAAs C40F60 and C45F55 exhibit relatively favorable cylinder compressive strengths with cement contents of 40% and 45%, respectively. Nonetheless, these strengths remain inferior to that attained by FAA C35F65 with a cement content of merely 35%. The utilization of higher cement content in FAAs not only fails to achieve optimal mechanical performance but also exacerbates carbon emissions, posing challenges to the sustainable development of the aggregate industry. Considering both carbon emissions and mechanical performance, it is determined that the optimal cement content for producing FAAs should not surpass 35%.

The results presented in Figure 4 indicate that the unconfined cylinder compressive strength of FAAs is notably high, ranging from 5.8 to 18.5 MPa. According to the national standard GB/T 17431.1 [27], materials with a strength exceeding 6.5 MPa are classified as high-strength LWAs suitable for the preparation of structural concrete. Hence, with the exception of C5F95, all FAAs meet the criteria for high-strength LWAs.

### 3.2. Micro-Structure of Fly Ash Aggregates

#### 3.2.1. XRD Patterns

**Figure 5:**
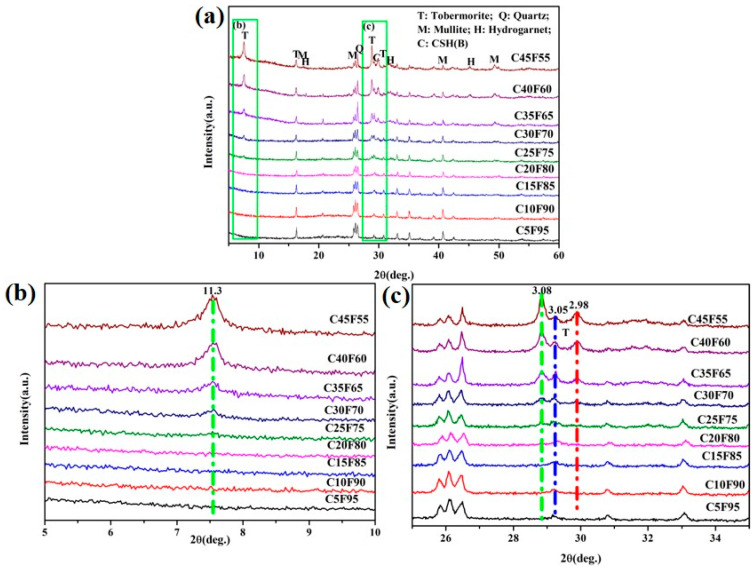
XRD patterns of FAAs. (**a**) phase composition; (**b**) FAAs specimen; (**c**) diffuse diffraction peak.

Results depicted in Figure 5a demonstrate that the phase composition of FAAs comprises residual mullite and quartz. In Figure 5c, a diffuse diffraction peak is discernible at 2θ = 29.2°, indicative of the presence of poorly crystalline hydration products, specifically amorphous C-S-H(B) (exhibiting characteristic peaks at 3.05 Å, corresponding to a composition range of 0.8~1.5 CaO·SiO_2_·nH_2_O). The data presented in Figure 5b,c reveal that as the cement content increases to 20 wt.%, the FAAs specimen C20F80 exhibits the formation of tobermorite, with main characteristic peaks at 11.3 Å, 3.08 Å, and 2.98 Å. Moreover, as illustrated in Figure 5a, an elevation in cement content to 25 wt% results in the emergence of hydrogarnet (3CaO·Al_2_O_3_·SiO_2_·4H_2_O, with primary characteristic peaks at 5.05 Å and 2.75 Å).

Consequently, for FAAs specimens ranging from C5F95 to C10F90, the predominant phases are the hydration product C-S-H(B), along with residual mullite and quartz. Specimens C15F85 to C20F80 primarily consist of the hydration product C-S-H(B), tobermorite, as well as residual mullite and quartz. In contrast, for FAAs specimens C25F75 to C45F55, their phase composition predominantly includes the hydration product C-S-H(B), tobermorite, hydrogarnet, alongside residual mullite and quartz. This observation underscores the significant influence of cement content on the types of hydration products present in FAAs, with increasing cement content leading to a greater diversity of hydration products.

Furthermore, the chemical composition analysis reveals that the SiO_2_ content in fly ash is 53.47%, while the CaO content is 2.98% (as shown in Table 1). In cement, the SiO_2_ content is 20.3% and the CaO content is 64% (also presented in Table 1). When the cement dosage is set at 45 wt.%, calculations based on the chemical composition of the raw materials indicate that the CaO/SiO_2_ ratio in FAAs is approximately 0.85, which is close to the ideal CaO/SiO_2_ ratio of tobermorite at 0.83. Theoretically, this suggests that calcium and silicon fully engage in reactions to form tobermorite. However, as evident in Figure 5a, the persistence of a diffraction peak for quartz in the C45F55 FAAs specimen indicates incomplete reaction of quartz and suggests an excess of Ca^2^⁺ due to an excessively high cement dosage of 45 wt.%.

As the cement content increases, the intensity of the diffraction peaks for mullite and quartz gradually diminishes (as depicted in Figure 5a), while the diffraction peak of C-S-H(B) becomes progressively broader (as shown in Figure 5c). This trend indicates that the incorporation of calcium sources promotes the formation of C-S-H(B), which subsequently reacts with silicate ions in solution to generate tobermorite. Consequently, tobermorite is observed in the FAAs specimen C20F80, and its diffraction peak intensity escalates with increasing cement content (as illustrated in Figure 5b,c). Furthermore, owing to the abundant presence of Al_2_O_3_ in FA, hydrogarnet emerges in the C25F75 specimen, with its diffraction peak intensity also augmenting as cement content rises (as demonstrated in Figure 5a). These findings collectively indicate that the increase in cement content significantly impacts the content of hydration products, as well as the quantities of mullite and quartz in the FAAs. Consequently, elevating the cement dosage effectively enhances the content of hydration products.

#### 3.2.2. Insoluble Matter

**Figure 6:**
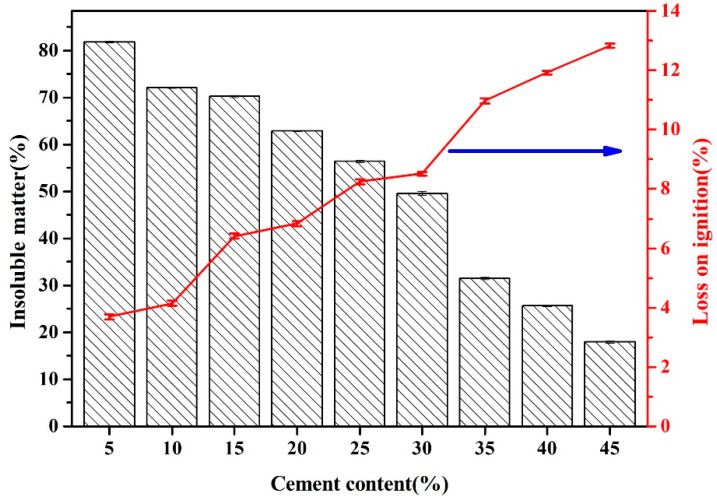
Insoluble matter and loss on ignition of FAAs. Note: the blue arrow indicates the supplementary y-axial.

Hydration products of FAAs (such as tobermorite and C-S-H gel) exhibit solubility in acidic environments, whereas substances like quartz and mullite remain refractory to acid dissolution. The proportion of materials that are resistant to hydrochloric acid dissolution is termed insoluble matter, with quartz and mullite constituting the predominant phases. These minerals act as a structural framework and significantly contribute to the mechanical strength of autoclaved products. Consequently, in adherence to the GB/T 12960-2019 [18], an assessment was conducted to determine the content of insoluble matter in FA, with the results illustrated in Figure 6.

A discernible pattern emerges: as the cement content progressively increases from 5 wt% to 10 wt.%, 15 wt.%, 20 wt.%, 25 wt.%, 30 wt.%, 35 wt.%, and ultimately reaches 40 wt% and 45 wt.%, the insoluble matter content of FAAs undergoes a decrease, transitioning from 81.88% to 72.09%, 70.24%, 62.86%, 56.37%, 49.58%, 31.56%, 25.72%, and finally to 17.96%. This decrease signifies a reduction in the residual quartz and mullite content within the FAAs with escalating cement dosage, which aligns with the observations depicted in Figure 5a, where the intensity of XRD diffraction peaks corresponding to mullite and quartz diminishes as cement content rises.

The quartz and mullite present within the insoluble matter serve as a structural scaffold for the FAAs, exerting a micro-aggregate effect. The interaction between micro-aggregate and filling effects significantly enhances the mechanical performance of composite materials by synergistically densifying the microstructure. Micro-aggregates, such as nanoparticles or silica fume, strengthen the matrix through interfacial bonding, pore refinement, and modulation of hydration products, reducing weak interfaces and limiting microcrack propagation. Simultaneously, the filling effect optimizes particle packing density across scales, minimizing porosity, distributing stress uniformly, and improving durability by reducing permeability. Together, these mechanisms create a hierarchical reinforcement: micro-aggregates address nanoscale voids, while fillers optimize mesoscale packing, leading to reduced total porosity, increased fracture energy via crack deflection, and synergistic strength gains beyond linear predictions. This interplay not only enhances compressive and flexural strength but also improves durability and sustainability by lowering cement content and CO_2_ emissions [28]. Consequently, this reduction in insoluble matter content leads to a diminution in the cylinder compressive strength when the cement content is augmented from 35 wt% to 45 wt.%. Nonetheless, it is noteworthy that as the cement content increases from 5 wt% to 35 wt.%, despite the downward trend in acid-insoluble matter (as illustrated in Figure 6), there is an augmentation of the micro-aggregate effects. Thus, the cylinder compressive strength of FAAs demonstrates an ascending trend (as depicted in Figure 6). To gain deeper insights into these observations, further analysis utilizing scanning electron microscopy (SEM) will be imperative.

As presented in Table 1, the SiO_2_ content in FA is 53.47%, whereas the CaO content is 2.98%. In contrast, cement exhibits a SiO_2_ content of 20.3% and a CaO content of 64%. When the cement proportion is varied from 5 wt% to 45 wt% in increments of 5 wt.%, the calculated CaO/SiO_2_ ratios of the raw materials in FAAs, based on their respective chemical compositions, range from 0.12 to 0.85, specifically 0.12, 0.19, 0.27, 0.35, 0.43, 0.52, 0.62, 0.73, and 0.85. Furthermore, as detailed in Table 3, the CaO/SiO_2_ ratios of the hydration products in these aggregates, determined from the insoluble residue content, span from 0.69 to 1.04, with specific values of 0.69, 0.69, 0.90, 0.93, 0.99, 1.04, 0.91, 0.98, and 1.03. At cement contents of 5 wt% and 10 wt.%, the CaO/SiO_2_ ratio of the hydration products is consistently measured at 0.69. This observation leads to the conclusion that the FAAs C5F95 and C10F90 should contain hydration products with a CaO/SiO2 ratio exceeding 0.69. A CaO/SiO_2_ ratio greater than 0.69, specifically within the range of 0.8 to 1.5 CaO·SiO_2_·nH_2_O, is indicative of the presence of CSH(B), as corroborated by the XRD pattern presented in Figure 5. For cement contents ranging from 15 wt% to 45 wt.%, the CaO/SiO_2_ ratios of the hydration products vary between 0.90 and 1.04, with a notable maximum value of 1.03 observed at a cement content of 45%. In the case of C45F55, it is anticipated that the CaO/SiO_2_ ratio of its hydration product will fluctuate around this maximum value of 1.03. The ideal CaO/SiO_2_ ratio for tobermorite is 0.83, while the CaO/SiO_2_ ratio for CSH(B) falls within the range of 0.8 to 1.5. Consequently, it can be reasonably inferred that both tobermorite and CSH(B) are likely present in the FAA C45F55, a conclusion supported by the XRD patterns depicted in Figure 5.

Based on the data presented in Figure 5, the primary constituents of FAAs include quartz, mullite, tobermorite, and hydrogarnet. Quartz and mullite exhibit comparatively stable physico-chemical properties, refraining from generating high-temperature volatile compounds. Tobermorite and hydrogarnet, on the other hand, harbor variable quantities of chemically bound water and hydroxyl groups. Below 300 °C, these hydrated compounds undergo dehydration, whereas at 750 °C, they experience dehydroxylation. Therefore, during the calcination process of FAAs at 900 °C conducted in this study, significant mass shifts occurred as a result of both dehydroxylation and dehydration. To indirectly infer the hydrated compound content, the loss on ignition (LOI) method was adopted to quantify the chemically bound water present.

**Figure 7:**
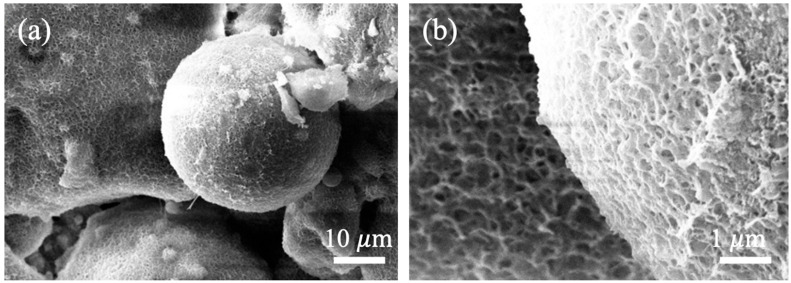
Micrographs of FAA C5F95: (**a**) micrograph with a larger scale; (**b**) micrograph with a smaller scale.

The findings from the LOI measurements are delineated in Figure 7. As the cement proportion escalates from 5 wt% to 45 wt.%, with incremental steps of 5 wt.%, the LOI of FAAs progressively climbs from 3.69% to 12.84%. This progression suggests that the hydrated compound content in FAAs augments with the increasing incorporation of cement. This observation aligns with the trend visible in Figure 5, wherein the intensity of XRD diffraction peaks corresponding to hydrated compounds intensifies with greater cement content. Moreover, this augmentation in hydrated compound content contributes to pronounced filling effects, ultimately elevating the density of FAAs and diminishing water absorption (refer to Figure 2). Concurrently, there is a discernible surge in apparent density (refer to Figure 3), while the cylinder compressive strength gradually amplifies as cement content increases from 5 wt% to 35 wt% (refer to Figure 4).

#### 3.2.3. Microstructure of Autoclaved Aggregates

The scanning electron microscopy (SEM) images of FAA C5F95 are exhibited in Figure 7. Panels (a) and (b) of Figure 7 reveal distinct spherical fly ash particles, with pores ranging from approximately 2 to 3 μm in length discernible between these particles. This observation suggests a scarcity of hydrated compounds and a diminished filling efficacy, giving rise to numerous micro-defects within the FAA and consequently, a less dense structural configuration. As a result, the water absorption rate of FAA C5F95 emerges as the highest (refer to Figure 2), its apparent density stands as the lowest (refer to Figure 3), and its cylinder compressive strength remains at a nadir (refer to Figure 4). Additionally, the exterior of the spherical FAA bears a rough texture, coated by a delicate stratum of flaky calcium silicate hydrate that weaves an interconnected mesh atop the spherical particles. This phenomenon transpires due to the elevated solubility and reactivity of Ca^2^⁺ ions, which engage in hydrothermal synthesis reactions on the FA bead surfaces, generating calcium silicate hydrate.

**Figure 8:**
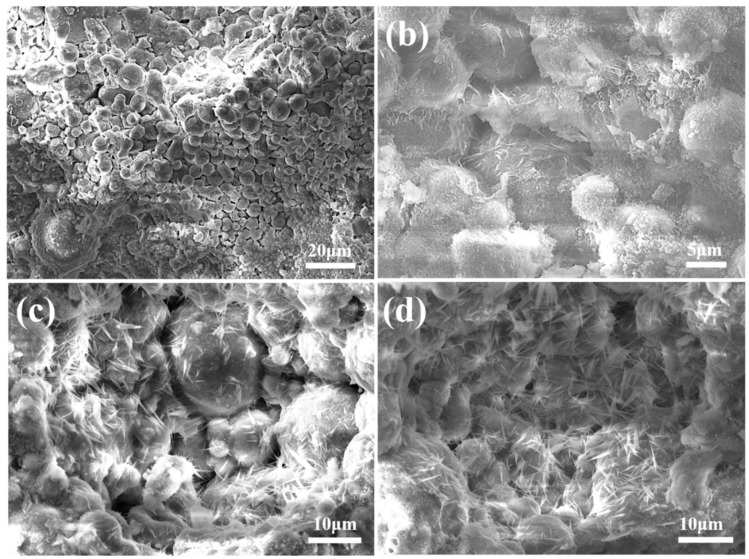
SEM images of FAAs: (**a**) C10F90; (**b**) C15F85; (**c**,**d**) C20F80.

The SEM images of the fly ash aggregates C10F90, C15F85, and C20F80 are exhibited in Figure 8. Figure 8a reveals a considerable amount of pores, ranging in size from 2 to 3 μm. However, in Figure 8b, a substantial diminution in the pore sizes is evident. Furthermore, Figure 8c,d, representing C20F80, exhibit a further diminution in pore size, with diameters predominantly between 1 and 2 μm. This trend suggests that an augmentation in cement proportion escalates the generation of hydration products, which exerts a filling effect that refines pore diameter and augments the density of the FAAs. Subsequently, this phenomenon contributes to a decrement in water absorption, as exemplified in Figure 2, an increment in apparent density, as shown in Figure 3, and an elevation in cylinder compressive strength, as demonstrated in Figure 4. Moreover, Figure 8c,d distinctly showcase the emergence of minuscule needle-like hydration products proliferating on the surface of spherical FAAs or interspersed among fly ash particles. As per the X-ray diffraction analysis presented in Figure 5, these hydration products are recognized as tobermorite.

**Figure 9:**
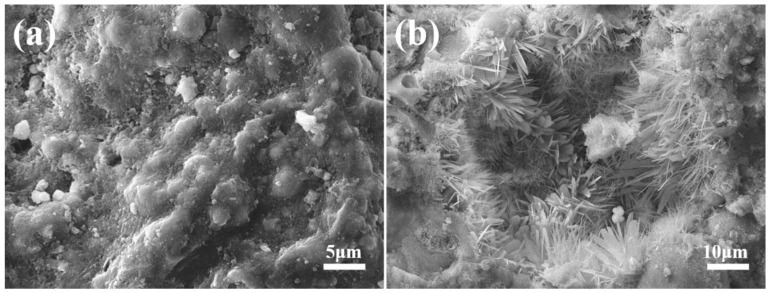
SEM images of FAA C25F75: (**a**) micrograph with a smaller scale; (**b**) micrograph with a larger scale.

The SEM image of the C25F75 fly ash aggregate is exhibited in Figure 9. Upon examination of Figure 9a, it becomes apparent that the pores amidst spherical fly ash particles are no longer discernible, as the surfaces of these spherical particles are now concealed by hydration products, which have also occupied the interstitial spaces. Solely spherical protrusions persist, signifying that the hydration products formed with a cement proportion of 25 wt% are adequate to occupy the voids, thereby mitigating the initial micro-defects inherent in the FAA. Additionally, Figure 9b distinctly reveals congregations of needle-like tobermorite, and their preponderance has escalated notably when juxtaposed with C20F80 (refer to Figure 8c,d). This finding concurs with the augmented diffraction peaks of tobermorite presented in Figure 5 and the amplified loss on ignition content illustrated in Figure 6.

**Figure 10:**
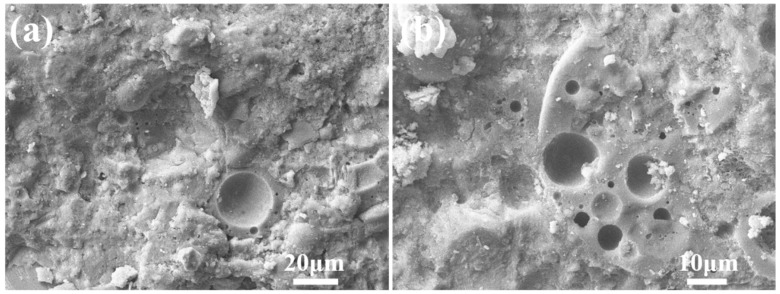
SEM images of FAA C35F65: (**a**) micrograph with a larger scale; (**b**) micrograph with a smaller scale.

The SEM images of the fly ash aggregate C35F65 are presented in Figure 10. Upon close examination of Figure 10a, it becomes evident that the proportion of spherical fly ash particles is relatively low, with only a scarce amount of these particles discernible. This observation suggests that a considerable quantity of hydration products has effectively occupied the pores and enveloped the surfaces of the spherical FA particles or filled the interstitial spaces between them. Furthermore, in Figure 10b, it is noticeable that the spherical FA particles are firmly integrated within the adjacent matrix, giving rise to a highly compacted structure. This cohesive structure facilitates the achievement of a maximum cylinder compressive strength of 18.5 MPa for the FAA C35F65, as documented in Figure 4.

**Figure 11:**
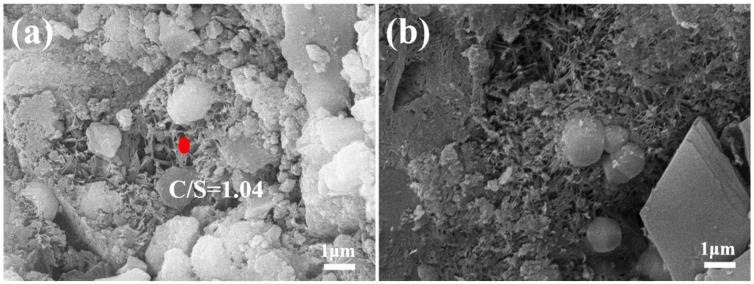
SEM images of FAA C45F55: (**a**) distinct flaky hydrates–CSH(B); (**b**) hydrogarnet.

At 180 °C, the solubility of Ca(OH)_2_ varies between 0.1 to 0.15 g/L, whereas glassy silica gel solubility can attain levels of 0.7 to 0.8 g/L. The dissolved Ca(OH)_2_ and SiO_2_ engage in hydrothermal reactions, leading to the synthesis of CSH(B) (xCaO·SiO₂·nH₂O, where x = 0.8~1.5). The heterogeneous nucleation of CSH(B) gives rise to the formation of tobermorite (5CaO·6SiO₂·5H₂O). The microstructure of FAA C45F55 was examined, and the results are exhibited in Figure 11a,b. Distinct flaky hydrates are visible in Figure 11, intersecting to forge a network structure. EDS analysis, conducted on these flaky hydrates (refer to Figure 11a), disclosed a CaO/SiO_2_ ratio approximating 1.04. This ratio suggests that the hydration product is, indeed, CSH(B). Assuming the engagement of all Ca^2^⁺ ions present in FAA C45F55 in the hydrothermal synthesis reaction, and considering its insoluble matter content (refer to Figure 6, 17.96 wt.%), it is computable that the calcium-to-silicon ratio for hydration products within the aggregate is approximately 1.03. This CaO/SiO_2_ ratio, exceeding the ideal tobermorite ratio of approximately 0.83, implies that besides tobermorite, there exists CSH(B) with a CaO/SiO_2_ ratio greater than 0.83 within FAA C45F55. Hence, SEM-EDS analysis substantiates the findings derived from insoluble matter examination.

The cement hydration process leads to the generation of C-S-H and Ca(OH)_2_. The precipitation of Ca(OH)_2_ occurs due to the reaction of calcium ions and hydroxide ions in the solution, and this precipitation nucleation aids in the formation of hydrated calcium silicate. In concrete, Ca(OH)_2_ manifests in diverse morphological forms, predominantly exhibiting a flaky structure. As evident in Figure 11a, the CSH(B) within FAAs showcases a flaky structure. This phenomenon arises from the dissolution of Ca(OH)_2_, releasing calcium ions that subsequently interact with silicate ions on the surface layers of Ca(OH)_2_ through hydration reactions, resulting in the in situ formation of flaky hydrated calcium silicate. Furthermore, Figure 11b reveals the emergence of a novel crystal, distinguished by its octahedral shape and granular morphology. These grains, being relatively coarse and exhibiting pronounced crystallinity, are identified as hydrogarnet, corroborated by the XRD analysis presented in Figure 5.

### 3.3. Mechanism of Strength Development in Fly Ash Aggregates

The aforementioned research reveals that the cylinder compressive strength of FAAs exhibits an initial increase followed by a subsequent decrease, peaking at a cement content of 35 wt.%. Through a comprehensive analysis encompassing insoluble matter content, loss on ignition content, mineralogical composition, and microstructure (as illustrated in Figure 5, Figure 6, Figure 7, Figure 8, Figure 9, Figure 10 and Figure 11), coupled with the evaluation of the cylinder compressive strength depicted in Figure 4, it becomes evident that at a cement content of 5 wt.%, FAAs display adequate micro-aggregate effects. Nevertheless, due to the absence of substantial hydration product filling effects, notable voids persist between FA particles, resulting in a porous microstructure that impedes the achievement of optimal cylinder compressive strength. This underscores that the mere presence of micro-aggregate effects does not ensure maximal cylinder compressive strength in FAAs.

Within the cement content range of 40% to 45%, despite the ample filling effect contributed by hydration products, the diminution of micro-aggregate effects leads to a reduction in cylinder compressive strength. This observation suggests that the presence of hydration product filling effects, in isolation, is insufficient for achieving peak cylinder compressive strength in FAAs. At a cement content of 35 wt.%, however, the cylinder compressive strength of FAAs attains its maximum value of 18.5 MPa. This optimum strength is facilitated by a combination of micro-aggregate effects and enhanced hydration product filling effects. This finding underscores the necessity for both micro-aggregate and filling effects to coexist for FAAs to reach their optimal cylinder compressive strength. Based on this analysis, a synergistic relationship is postulated between micro-aggregate and filling effects. It is only through their concerted action that FAAs can achieve superior cylinder compressive strengths. This synergistic effect offers deeper insights into the mechanisms underlying the excellent mechanical properties observed under optimal cement content conditions for these FAAs.

## 4. Conclusions

FA, an industrial waste, has been effectively repurposed by mixing it directly with cement to produce FAAs through pelletizing and autoclaving techniques. The research findings yield the following conclusions.

(1) As cement content increases from 5% to 35%, the filling effect of hydration products improves, progressively boosting the cylinder compressive strength of FAAs. However, beyond 35% cement content, the micro-aggregate effect diminishes, leading to a decrease in strength. The peak compressive strength of 18.5 MPa is achieved at 35% cement content.

(2) The increase in cement quantities not only enhances the formation of hydration products but also augments the filling effect within the FAAs. This subsequently leads to a reduction in water absorption and an increase in the apparent density of the FAAs. The optimal balance between these factors is achieved at a specific cement content, resulting in improved overall performance characteristics of the FAAs.

(3) FAAs exhibit water absorption rates between 2.07% and 12.88% after 1 h, and between 4.74% and 17.79% after 24 h. The apparent density of FAAs ranges from 1536 kg/m^3^ to 1946 kg/m^3^, satisfying lightweight criteria. Compressive strength varies from 8.7 MPa to 18.5 MPa when cement content is adjusted between 10% and 45%, aligning with high-strength LWA standards specified in the national standards.

Overall, fly ash aggregates demonstrate distinct advantages and limitations compared to other lightweight aggregates like expanded shale, clay, slate, or natural volcanic aggregates (e.g., pumice). They exhibit superior mechanical strength and cost-efficiency compared to many LWAs, driven by their pozzolanic reactivity and micro-filling capacity. However, their higher water absorption and moderate density limit applications requiring extreme thermal insulation. For structural lightweight concretes (e.g., 1600–2000 kg/m^3^ density), FAAs outperform expanded shale/clay by 20–30% in compressive strength while maintaining comparable durability. Natural pumice remains superior for ultra-lightweight insulative applications but at higher cost and lower strength. The numerical trade-offs position FAA as a sustainable middle-ground solution for balanced mechanical and thermal performance.

## Figures and Tables

**Table 1:** Primary chemical compositions of raw materials (%).

Component	SiO_2_	Al_2_O_3_	Fe_2_O_3_	CaO	MgO	K_2_O
Fly ash	53.47	30.48	4.73	2.94	0.95	-
Cement	20.30	5.61	3.25	64.00	1.17	-

**Table 2:** Mix proportions of fly ash aggregates (FAAs) (wt.%).

Notation	Core		Shell	Thickness
	Cement	Fly Ash	Fly Ash/Cement	Shell/Core
C5F95	5	95	85:15	4
C10F90	10	90	85:15	4
C15F85	15	85	85:15	4
C20F80	20	80	85:15	4
C25F75	25	75	85:15	4
C30F70	30	70	85:15	4
C35F65	35	65	85:15	4
C40F60	40	60	85:15	4
C45F55	45	55	85:15	4

**Table 3:** The CaO/SiO_2_ ratios of and insoluble matter of FAAs.

Notation	Ca/Si ^a^	R (wt.%)	Ca/Si ^b^
C5F95	0.12	81.88	0.69
C10F90	0.19	72.09	0.69
C15F85	0.27	70.24	0.90
C20F80	0.35	62.86	0.93
C25F75	0.43	56.37	0.99
C30F70	0.52	49.58	1.04
C35F65	0.62	31.56	0.91
C40F60	0.73	25.72	0.98
C45F55	0.85	17.96	1.03

Note: Ca/Si ^a^—the CaO/SiO_2_ ratio of the raw material; Ca/Si ^b^—the CaO/SiO_2_ ratio of the hydration products; R—insoluble matter; Ca/Si ^b^ = Ca/Si ^a^/(1 − R).

## Data Availability

The original contributions presented in this study are included in the article. Further inquiries can be directed to the corresponding authors.
